# Sex‐Specific Associations Between Prebiotic Supplement Intake and Sarcopenia Risk: Evidence From NHANES

**DOI:** 10.1002/fsn3.70410

**Published:** 2025-07-25

**Authors:** Wenming Song, Jing Zhang, Yongxin Sha, Weixuan Liu, Changbo Hu

**Affiliations:** ^1^ Department of Chinese and Western Medicine Combined Changde Hospital, Xiangya School of Medicine, Central South University (The First People's Hospital of Changde City) Changde China; ^2^ Department of Ophthalmology Changde Hospital, Xiangya School of Medicine, Central South University (The First People's Hospital of Changde City) Changde China

**Keywords:** muscle health, NHANES, prebiotic supplements, sarcopenia, sex‐specific effects

## Abstract

Sarcopenia, characterized by the progressive loss of skeletal muscle mass and strength, poses a significant public health challenge. This study explores the sex‐specific associations between prebiotic intake and the risk of sarcopenia, utilizing data from the National Health and Nutrition Examination Survey (NHANES) conducted between 2011 and 2014. Adult participants provided information on their prebiotic consumption and sarcopenia status, which was defined according to the Foundation for the National Institutes of Health (FNIH) criteria, focusing on grip strength and appendicular skeletal muscle mass adjusted for body mass index (BMI). The analysis identified 4306 individuals as nonconsumers of prebiotics, whereas 157 were identified as consumers. The results showed an inverse association between prebiotic intake and the risk of sarcopenia among females, with an odds ratio of 0.11 (95% CI: 0.05–0.32, *p* = 0.01). In contrast, no significant association was observed in males. These findings suggest that prebiotic consumption may be particularly beneficial in reducing the risk of sarcopenia among adult women, highlighting the need for further research to investigate the underlying mechanisms.

## Graphical abstract

The study found that prebiotic intake is significantly associated with a reduced risk of sarcopenia in adult women but not in men, based on data from NHANES 2011–2014 using FNIH criteria. After adjusting for multiple confounders, female prebiotic consumers had lower odds of sarcopenia, while no significant association was observed in males. These results suggest potential sex‐specific benefits of prebiotic consumption for muscle health and highlight the need for further research on underlying mechanisms.

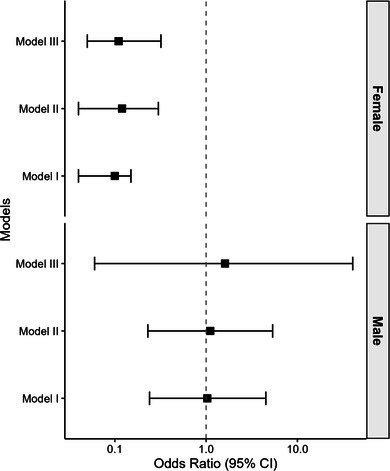

## 1 Introduction

Sarcopenia, a progressive loss of skeletal muscle mass and strength, is a significant public health concern affecting millions worldwide. While it predominantly affects older adults, its prevention and management are relevant across various age groups (Liu et al. 2023; Wang et al. 2022). It is defined by various criteria, including those set by the Foundation for the National Institutes of Health (FNIH) and the European Working Group on Sarcopenia in Older People (EWGSOP) (Cruz‐Jentoft et al. 2019; Studenski et al. 2014). The FNIH criteria define sarcopenia based on weakness and low muscle mass (Studenski et al. 2014). In contrast, the EWGSOP criteria emphasize low muscle strength as a primary indicator, with muscle quantity and quality used to confirm the diagnosis, and physical performance measures to assess severity (Cruz‐Jentoft et al. 2019). Recent research has highlighted the potential role of the gut microbiota in sarcopenia development, with alterations in gut microbiota composition influencing muscle health. Prebiotics, which selectively promote beneficial gut bacteria, have shown promise in improving muscle mass in sarcopenic patients (Tominaga et al. 2021).

Interestingly, the composition of the gut microbiome is known to differ between men and women, with several bacterial genera like Bacteroides, Veillonella, Methanobrevibacter, and Bilophila exhibiting sex‐based variations (Fransen et al. 2017; Yoon and Kim 2021). These differences may influence how prebiotics modulate muscle health, as prebiotics selectively promote beneficial bacteria like Bifidobacteria, which have been linked to reduced sarcopenia risk in women (Wang et al. 2023). Additionally, sex hormones play a significant role in muscle health, with evidence suggesting that hormonal influences can affect muscle protein synthesis and breakdown differently in men and women (Brown 2008). Furthermore, emerging evidence suggests that the effects of prebiotics, which serve as nutrients for probiotic gut bacteria, may also be sex‐specific (Jiang et al. 2023; Kadyan et al. 2023). Some studies have reported that prebiotic consumption appears to be more effective in modulating gut microbiota and conferring health benefits in females compared to males (Jiang et al. 2023).

However, it remains unclear whether prebiotic supplementation exhibits sex‐specific associations with sarcopenia risk reduction. Given the potential links between gut microbiota, prebiotics, and muscle health, this study aims to investigate whether the relationship between prebiotic intake and sarcopenia risk differs by sex using data from the National Health and Nutrition Examination Survey (NHANES).

## 2 Methods

### 2.1 Data Source and Study Population

This analysis utilized data from the NHANES. The study sample included adult men and women aged 18 years or older who had available data on prebiotic intake, sarcopenia status, and relevant covariates. The NHANES 2011–2014 cycle was specifically used as it was the only recent dataset containing the necessary sarcopenia assessment measures required for this analysis. The National Center for Health Statistics (NCHS) Research Ethics Review Board approved the NHANES data collection protocols, and documented consent was obtained from participants.

### 2.2 Assessment of Prebiotic Intake

Prebiotic intake was assessed using data from the Dietary Supplement Questionnaire (DSQ) and medication records incorporated into the NHANES surveys. All dietary supplement names, dietary supplement ingredients, as well as medication names and medication ingredients from these databases were text‐mined for key phrases to identify products containing prebiotics. Specifically, the text data was systematically searched for terms like “inulin,” “oligos,” and other known prebiotic compounds. Any products with these ingredients listed were classified as prebiotic sources.

### 2.3 Measurements of Covariates

Several covariates were obtained from the NHANES database and considered potential confounders based on known or suspected relationships with prebiotic intake, sarcopenia risk, or both. Demographic factors included age, sex, and self‐identified ethnicity. Socioeconomic characteristics comprised the poverty‐income ratio (PIR) and educational levels. Anthropometric data included body mass index (BMI). Lifestyle attributes included physical activity level based on self‐reported minutes of moderate and vigorous activity per week.

### 2.4 Definition of Sarcopenia

In this study, sarcopenia was defined according to the FNIH Sarcopenia Project criteria (Studenski et al. 2014), which include weakness and muscle loss as the primary factors for diagnosis. Grip strength and appendicular skeletal muscle mass (ASM) were used as quantitative indicators for weakness and muscle loss, respectively. Both grip strength and ASM were adjusted for BMI to account for body size differences. The adjusted grip strength and ASM were termed grip strength_BMI_ and ASM_BMI_, respectively. The cutoff values for grip strength_BMI_ were < 1.0 for males and < 0.56 for females. For ASM_BMI_, the cutoff values were < 0.789 for males and < 0.512 for females. Participants who met both the grip strength_BMI_ and ASM_BMI_ cutoff criteria were classified as having sarcopenia.

### 2.5 Statistical Analyses

All statistical analyses were performed using R version 4.3.0 (http://www.R‐project.org, The R Foundation). Descriptive statistics were calculated to summarize the baseline characteristics of the study population. Continuous variables were reported as means (standard deviations), while categorical variables were presented as numbers (percentages). Differences in baseline characteristics between prebiotic consumers and nonconsumers were assessed using independent sample *t*‐tests for continuous variables and chi‐square tests for categorical variables. Univariate logistic regression analyses were conducted to examine the crude associations between potential risk factors and sarcopenia. To investigate potential effect modification, stratified analyses were performed by sex, education level, and ethnicity. Logistic regression models were constructed within each stratum to assess the association between prebiotic intake and sarcopenia risk. After adjusting for relevant confounders, multivariate logistic regression analyses were then employed to evaluate the independent relationship between prebiotic consumption and sarcopenia. Three models with progressive adjustment were built. Model I was unadjusted. Model II was adjusted for age, sex, and BMI. Model III was additionally adjusted for PIR, education, ethnicity, and physical activity. All regression analyses accounted for the complex survey design and sample weights of NHANES using appropriate techniques. A two‐tailed *p* < 0.05 was considered statistically significant for all analyses.

## 3 Results

### 3.1 Characteristics of Participants

The study included 4306 nonconsumers of prebiotics (44.7% male) and 157 prebiotic consumers (37.9% male). As shown in Table 1, prebiotic consumers were significantly older than nonconsumers, with mean ages of 41.65 ± 1.31 and 36.86 ± 0.40 years, respectively (*p* < 0.001). There was no significant difference in sex distribution between the two groups (*p* = 0.17). Prebiotic consumers had a significantly higher mean PIR than nonconsumers, indicating higher socioeconomic status (3.51 vs. 3.13, *p* = 0.02). While prebiotic consumers tended to have lower physical activity levels, this difference was not statistically significant. There was a significant difference in education level between the two groups (*p* = 0.03), with a higher proportion of prebiotic consumers being college graduates than nonconsumers (41.4% vs. 32.7%). The distribution of ethnicity categories did not differ significantly between consumers and nonconsumers. Finally, the prevalence of sarcopenia did not differ significantly between consumers and nonconsumers (*p* = 0.66).

**TABLE 1:** Characteristics of consumers and nonconsumers of prebiotics.

Variable	Nonconsumers	Consumers	*p*
Age, years [mean (SE)]	36.86 (0.40)	41.65 (1.31)	< 0.001
Sex [*n* (%)]			
Male	2008 (44.69)	66 (37.89)	0.17
Female	2545 (55.31)	91 (62.11)	
BMI, kg/m^2^ [mean (SE)]	27.65 (0.19)	28.48 (0.88)	0.31
PIR [mean (SE)]	3.13 (0.09)	3.51 (0.16)	0.02
Physical activity, MET [mean (SE)]	4261.23 (146.34)	3712.00 (439.89)	0.24
Education level [*n* (%)]			
Less than 9th grade	902 (12.13)	15 (6.23)	0.03
9th–11th grade	579 (9.49)	10 (4.01)	
High school graduate	680 (16.18)	31 (20.18)	
College	1197 (29.47)	49 (28.16)	
College graduate	1193 (32.74)	52 (41.42)	
Ethnicity [*n* (%)]			
Non‐Hispanic White	1862 (69.32)	69 (72.02)	0.7
Non‐Hispanic Black	880 (9.24)	27 (7.23)	
Hispanic	855 (12.28)	32 (12.86)	
Other	956 (9.17)	29 (7.89)	
Sarcopenia [*n* (%)]			
No	4132 (97.33)	148 (98.02)	0.66
Yes	174 (2.67)	4 (1.98)	

Abbreviations: BMI, body mass index; MET, metabolic equivalent tasks; PIR, poverty‐income ratio.

### 3.2 Univariate Analysis

Univariate logistic regression analysis was performed to examine the association between potential risk factors and sarcopenia. As shown in Table 2, increasing age was associated with higher odds of sarcopenia (OR: 0.95, 95% CI: 0.92–0.97, *p* < 0.0001). Females had significantly lower odds of sarcopenia compared to males (OR: 0.12, 95% CI: 0.07–0.21, *p* < 0.0001). Compared to those with less than 9th‐grade education, higher education levels were associated with lower odds of sarcopenia, with *ORs* ranging from 0.06 for college graduates (95% CI: 0.03–0.13, *p* < 0.0001) to 0.12 for the 9th–11th grade (95% CI: 0.05–0.28, *p* < 0.0001). Prebiotic intake was not significantly associated with sarcopenia in univariate analysis.

**TABLE 2:** Univariate analysis.

Variables	OR (95% CI)	*p*
Age, years	0.95 (0.92, 0.97)	< 0.0001
Sex		
Male	Ref	Ref
Female	0.12 (0.07, 0.21)	< 0.0001
BMI, kg/m^2^	1.04 (1.00, 1.08)	0.05
PIR	0.88 (0.76, 1.01)	0.07
Education level		
Less than 9th grade	Ref	Ref
9th–11th grade	0.12 (0.05, 0.28)	< 0.0001
High school graduate	0.06 (0.02, 0.16)	< 0.0001
College	0.10 (0.04, 0.27)	< 0.0001
College graduate	0.06 (0.03, 0.13)	< 0.0001
Ethnicity		
Non‐Hispanic White	Ref	Ref
Non‐Hispanic Black	0.98 (0.59, 1.61)	0.92
Hispanic	1.49 (0.89, 2.50)	0.13
Other	1.88 (1.06, 3.33)	0.03
Prebiotic		
Nonconsumers	Ref	Ref
Consumers	0.74 (0.18, 3.01)	0.66
Physical activity, MET	1.00 (1.00, 1.00)	0.2

Abbreviations: BMI, body mass index; MET, metabolic equivalent tasks; PIR, poverty‐income ratio.

### 3.3 Stratified Analysis

We conducted stratified analyses by sex, education level, and ethnicity to examine whether the association between prebiotic intake and sarcopenia differed across these subgroups. As shown in Table 3, the OR for the association between prebiotic intake and sarcopenia reduction was 1.033 (95% CI: 0.237–4.508; *p* = 0.965) among males and 0.10 (95% CI: 0.04–0.15; *p* < 0.001) among females. This suggests that while prebiotic intake was not associated with sarcopenia reduction in males, there was an association in females. When stratified by education level, those with less than 9th‐grade education had an OR of 0.277 (95% CI: 0.032–2.408; *p* = 0.235) for the association between prebiotics and sarcopenia reduction. The ORs for those who were high school graduates, those with some college, and college graduates were 1.717 (*p* = 0.631), 2.888 (*p* = 0.330), and 0.637 (*p* = 0.699), respectively. Finally, when examining ethnicity, non‐Hispanic whites had an OR of 0.862 (*p* = 0.868), non‐Hispanic blacks had an OR of 1.768 (*p* = 0.600), and Hispanics had an OR of 0.529 (*p* = 0.534).

**TABLE 3:** Stratified analysis.

Variables	OR (95% CI)		*p*
	Nonconsumers	Consumers	
Sex			
Male	Ref	1.033 (0.237, 4.508)	0.965
Female	Ref	0.10 (0.04, 0.15)	< 0.001
Education level			
Less than 9th grade	Ref	0.277 (0.032, 2.408)	0.235
9th–11th grade	Ref	—§	—§
High school graduate	Ref	1.717 (0.177, 16.614)	0.631
College	Ref	2.888 (0.325, 25.663)	0.330
College graduate	Ref	0.637 (0.060, 6.751)	0.699
Ethnicity			
Non‐Hispanic White	Ref	0.862 (0.142, 5.240)	0.868
Non‐Hispanic Black	Ref	1.768 (0.197, 15.896)	0.600
Hispanic	Ref	0.529 (0.067, 4.193)	0.534
Other	Ref	—§	—§

—§: Logistic regression not applicable due to zero count in this category.

### 3.4 Multivariate Analysis

After adjusting for potential confounders, we conducted multivariate logistic regression analyses to examine the association between prebiotic intake and sarcopenia risk (Table S1). Three models with progressive degrees of adjustment were constructed. Model I was unadjusted; Model II was adjusted for age, sex, and BMI; and Model III was further adjusted for other covariates, including PIR, education level, ethnicity, and physical activity. The relationship between prebiotic intake and sarcopenia was not statistically significant in all models.

Table S2 shows the association between prebiotic intake and sarcopenia risk in male individuals from three logistic regression models with progressive levels of adjustment. In the unadjusted model (Model I), prebiotic consumption was not significantly associated with sarcopenia risk compared to nonconsumption (OR: 1.03, 95% CI: 0.24–4.51, *p* = 0.96). The association was similarly nonsignificant after adjusting for age and BMI (Model II) (OR: 1.11, 95% CI: 0.23–5.35, *p* = 0.89). Finally, with further adjustment for socioeconomic and lifestyle factors (Model III), prebiotic intake remained unrelated to sarcopenia (OR: 1.61, 95% CI: 0.06–40.31, *p* = 0.76). Thus, prebiotic consumption does not appear to be independently associated with reduced odds of sarcopenia in men based on this analysis.

Table 4 shows the association between prebiotic intake and sarcopenia risk in female individuals from three logistic regression models with progressive levels of adjustment. In the unadjusted model (Model I), female prebiotic consumers had significantly lower odds of sarcopenia compared to nonconsumers (OR: 0.10, 95% CI: 0.04–0.15, *p* < 0.001). This strong protective association persisted after adjusting for age and BMI (Model II, OR: 0.12, 95% CI: 0.04–0.30, *p* < 0.001) as well as other covariates including income, education, ethnicity, and physical activity (Model III, OR: 0.11, 95% CI: 0.05–0.32, *p* = 0.01). Thus, prebiotic consumption exhibited an inverse association with sarcopenia among females.

**TABLE 4:** The relationship between prebiotics and sarcopenia in female individuals.

Variables	Model I (OR 95% CI, *p*)	Model II (OR 95% CI, *p*)	Model III (OR 95% CI, *p*)
Prebiotics			
Nonconsumers	Ref	Ref	Ref
Consumers	0.10 (0.04, 0.15) < 0.001	0.12 (0.04, 0.30) < 0.001	0.11 (0.05, 0.32) 0.01

*Note:* Model I does not adjust for other covariates; Model II adjusts for age and BMI; Model III adjusts for age, BMI, PIR, education level, ethnicity, and physical activity.

## 4 Discussion

This study used NHANES data to explore potential sex differences in the relationship between prebiotic intake and sarcopenia risk. Our results suggest that prebiotic consumption may be associated with reduced odds of sarcopenia among females but not males.

In univariate analysis, we did not find a significant crude association between prebiotic intake and sarcopenia. However, in stratified analyses, prebiotic consumers had significantly lower odds of sarcopenia compared to nonconsumers in the female subgroup. This protective association persisted after extensive adjustment for confounders in logistic regression models. In contrast, prebiotic intake remained unrelated to sarcopenia risk in males regardless of adjustments.

Our findings suggest a potential sex‐specific relationship between prebiotic intake and sarcopenia risk reduction. While previous studies have explored links between gut microbiota, prebiotics, and muscle health, evidence directly comparing these associations across sexes has been limited (Fransen et al. 2017; Jiang et al. 2023; Kadyan et al. 2023). Bifidobacteria levels have been correlated with lower sarcopenia risk in elderly women, and prebiotics like 1‐kestose can increase bifidobacteria abundance while improving muscle mass in sarcopenic patients (Wang et al. 2023). However, the mechanisms underlying potential sex differences in how prebiotics may modulate sarcopenia development require further investigation. Our results indicate that prebiotic supplementation may preferentially benefit muscle health in women compared to men. Further research is needed to elucidate the gut–muscle axis and its sex‐specific nuances that could inform tailored nutritional interventions for sarcopenia management.

Potential mechanisms underlying this relationship require further elucidation but might involve sex hormones influencing gut microbiota and subsequent effects on muscle protein synthesis or breakdown (Yoon and Kim 2021). Additionally, prebiotics may reduce sarcopenia progression by modulating gut permeability, inflammation, and insulin resistance (Liu et al. 2023).

Our analysis has some limitations that warrant consideration. First, the cross‐sectional design restricts the ability to infer causality between prebiotic intake and sarcopenia risk. Additionally, self‐reported dietary data may be subject to recall biases, which could affect the accuracy of prebiotic intake assessments. Another limitation is the relatively small sample size of prebiotic consumers, particularly when stratified by sex. Furthermore, we did not directly control the intake of other dietary components, such as fiber, probiotics, or proteins, which influence muscle health. Future studies should aim to recruit larger cohorts and incorporate detailed dietary assessments to provide a more comprehensive understanding of how prebiotics interact with other nutrients in relation to sarcopenia risk. Despite these limitations, our findings highlight the potential for sex‐specific benefits of prebiotic intake in reducing sarcopenia risk, underscoring the need for further research to elucidate the underlying mechanisms.

In conclusion, this study found that prebiotic intake exhibits a robust inverse relationship with sarcopenia among adult women but not men using NHANES data. Our results highlight the need to consider sex differences when examining associations between nutrition, gut microbiota, and muscle health outcomes.

## Author Contributions

C.H. designed the study. W.S. wrote the manuscript. J.Z. and Y.S. analyzed the data. C.H. and W.L. reviewed and edited the manuscript. All authors read and approved the final manuscript.

## Ethics Statement

The studies involving humans were approved by the Research Ethics Review Board of the National Centre for Health Statistics (NCHS). The studies were conducted following the local legislation and institutional requirements. The participants provided their written informed consent to participate in this study.

## Conflicts of Interest

The authors declare no conflicts of interest.

## Supporting information


**Table S1.** Multivariate analysis.

**Table S2.** The relationship between prebiotics and sarcopenia in male individuals.

## Data Availability

The datasets generated and/or analyzed during the current study are available in the NHANES database, https://wwwn.cdc.gov/nchs/nhanes/.
