# Supplementary material for: Sex‐Specific Associations Between Prebiotic Supplement Intake and Sarcopenia Risk: Evidence From NHANES
Source: Food Sci Nutr. 2025 Jul 25;13(7):e70410. doi: 10.1002/fsn3.70410 (PMC12290476; doi:10.1002/fsn3.70410)
Supplement: Supplementary file 1 — Table S1. Multivariate analysis. [file FSN3-13-e70410-s001.docx]

**Table S1: Multivariate analysis**

| Variables | Model I (*OR* 95%*CI* *P*) | Model II (*OR* 95%*CI* *P*) | Model III (*OR* 95%*CI* *P*) |
| --- | --- | --- | --- |
| Prebiotics |  |  |  |
| Non-consumers | ref | ref | ref |
| Consumers | 0.74 (0.18,3.01) 0.66 | 0.95 (0.21,4.30) 0.94 | 0.76 (0.05,11.01) 0.83 |

Model I does not adjust for other covariance; Model II adjusts for age, sex and BMI; Model III adjusts for age, sex, BMI, PIR, education level, ethnicity, and physical activity.
