# Supplementary material for: Sex‐Specific Associations Between Prebiotic Supplement Intake and Sarcopenia Risk: Evidence From NHANES
Source: Food Sci Nutr. 2025 Jul 25;13(7):e70410. doi: 10.1002/fsn3.70410 (PMC12290476; doi:10.1002/fsn3.70410)
Supplement: Supplementary file 2 — Table S2. The relationship between prebiotics and sarcopenia in male individuals. [file FSN3-13-e70410-s002.docx]

**Table S2: The relationship between prebiotics and sarcopenia in male individuals**

| Variables | Model I (*OR* 95%*CI* *P*) | Model II (*OR* 95%*CI* *P*) | Model III (*OR* 95%*CI* *P*) |
| --- | --- | --- | --- |
| Prebiotics |  |  |  |
| Non-consumers | ref | ref | ref |
| Consumers | 1.03 (0.24,4.51) 0.96 | 1.11 (0.23,5.35) 0.89 | 1.61 (0.06,40.31) 0.76 |

Model I does not adjust for other covariance; Model II adjusts for age and BMI; Model III adjusts for age, BMI, PIR, education level, ethnicity, and physical activity.
